# On the Use of Surface Plasmon Resonance Biosensing to Understand IgG-FcγR Interactions

**DOI:** 10.3390/ijms22126616

**Published:** 2021-06-21

**Authors:** Catherine Forest-Nault, Jimmy Gaudreault, Olivier Henry, Yves Durocher, Gregory De Crescenzo

**Affiliations:** 1 Department of Chemical Engineering, Polytechnique Montreal, Montreal, QC H3T 1J4, Canada; catherine.forest-nault@polymtl.ca (C.F.-N.); jimmy.gaudreault@polymtl.ca (J.G.); olivier.henry@polymtl.ca (O.H.); 2 Human Health Therapeutics Research Centre, National Research Council of Canada, Montreal, QC H4P 2R2, Canada; yves.durocher@cnrc-nrc.gc.ca; 3 Department of Biochemistry and Molecular Medicine, University of Montreal, Montreal, QC H3T 1J4, Canada

**Keywords:** Fcγ receptors, surface plasmon resonance (SPR), monoclonal antibodies (mAbs), N-glycosylation, SPR data analysis

## Abstract

Surface plasmon resonance (SPR)-based optical biosensors offer real-time and label-free analysis of protein interactions, which has extensively contributed to the discovery and development of therapeutic monoclonal antibodies (mAbs). As the biopharmaceutical market for these biologics and their biosimilars is rapidly growing, the role of SPR biosensors in drug discovery and quality assessment is becoming increasingly prominent. One of the critical quality attributes of mAbs is the N-glycosylation of their Fc region. Other than providing stability to the antibody, the Fc N-glycosylation influences immunoglobulin G (IgG) interactions with the Fcγ receptors (FcγRs), modulating the immune response. Over the past two decades, several studies have relied on SPR-based assays to characterize the influence of N-glycosylation upon the IgG-FcγR interactions. While these studies have unveiled key information, many conclusions are still debated in the literature. These discrepancies can be, in part, attributed to the design of the reported SPR-based assays as well as the methodology applied to SPR data analysis. In fact, the SPR biosensor best practices have evolved over the years, and several biases have been pointed out in the development of experimental SPR protocols. In parallel, newly developed algorithms and data analysis methods now allow taking into consideration complex biomolecular kinetics. In this review, we detail the use of different SPR biosensing approaches for characterizing the IgG-FcγR interactions, highlighting their merit and inherent experimental complexity. Furthermore, we review the latest SPR-derived conclusions on the influence of the N-glycosylation upon the IgG-FcγR interactions and underline the differences and similarities across the literature. Finally, we explore new avenues taking advantage of novel computational analysis of SPR results as well as the latest strategies to control the glycoprofile of mAbs during production, which could lead to a better understanding and modelling of the IgG-FcγRs interactions.

## 1. Introduction

Surface plasmon resonance (SPR)-based biosensors have become a standard tool in the discovery and development pipelines of therapeutic monoclonal antibodies (mAbs). As the biopharmaceutical market for mAbs experienced exponential growth over the past two decades, the advantages of the SPR biosensor technique became increasingly relevant [1,2]. Real-time analysis, low consumption of unlabeled samples, automation, and mid-to-high throughput screening capabilities are some of the enabling features which make SPR biosensing a valuable tool in mAb discovery, antigen-antibody kinetics characterization, epitope profiling, and immunogenicity screening [2]. Moreover, SPR biosensors have also been integrated into the bioprocess and quality analysis streams as an efficient tool to quantify the mAbs’ critical quality attributes and optimize production processes [3]. Overall, the SPR-based biosensors are acknowledged to be a robust, easy-to-use, and versatile alternative to standard techniques such as ELISA [2].

Traction from the therapeutic mAb industry for cost- and time-efficient technologies is also growing fast due to the arrival of biosimilars and biobetters, now taking more and more shares of a market estimated to reach USD $300 billion by 2025 [2]. Indeed, while from 2002 to 2012, only 16 mAbs (and no biosimilars) were approved, 31 novel mAbs and 11 biosimilars were approved between 2013 and 2017 only [4]. New guidelines from regulatory agencies were created to help define the development and production of biosimilars [5]. There are two major axes that need to be addressed for the success of a biosimilar: characterization of the product and demonstration of its similarity with its reference [6]. However, antibodies are complex proteins which quality attributes are sensitive to not only the host cell platform used but all the steps of bioprocess manufacturing [7]. Various changes in production protocols can promote changes in the mAb structure and affect product function, pharmacokinetics, and pharmacodynamics (PK/PD) [8]. Post-translational modifications such as glycosylation can be altered, which in turn affect mAb immunogenicity and clearance. In that respect, glycosylation is often presented as one of the most critical quality attributes of the IgG subfamily of mAbs as it can also modulate their interactions with the Fc gamma receptors (FcγRs), those being responsible for the IgG effector functions such as the antibody-dependent cell-mediated cytotoxicity (ADCC) [9].

Along with the advances made in SPR-based analysis, several studies have investigated the interactions between immunoglobulin G (IgG) and the FcγRs in an effort to better understand the binding mechanism and the impact of IgG and FcγR glycosylation upon IgG-FcγR interactions [10,11,12]. In this review, we note key elements defining their interactions and discuss how SPR-based studies over the past two decades have helped quantify these bindings. As the results show a deeper complexity than a simple 1:1 Langmuir mechanism [13,14], we compare results and experimental approaches to understand the reported similarities and discrepancies. Based on a survey of the available literature, as well as our knowledge of the biology of the interactions, it is reasonable to think that the observed complexity is due to both artifactual and biological causes. Indeed, while one may question the design of some published SPR protocols, several studies reported unambiguous kinetic differences for various IgG glycoforms binding to FcγRs by SPR. As SPR-based analysis of IgG quality attributes is growing in the therapeutic mAb industry, it is important to identify possible biases and standardize the experimental approaches. At last, we discuss the latest platforms for controlled IgG production as well as novel approaches taking advantage of more complex computational analysis of the SPR results that could shed a novel light, in combination with SPR biosensing, on the IgG-FcγRs interactions.

## 2. IgG-FcγR Interaction

### 2.1. Therapeutic mAbs Quality Attributes

Eighty percent of therapeutic mAbs belong to the IgG1 type. IgG1 are composed of two Fab domains binding an antigen and one Fc domain that binds different types of Fc receptors to trigger effector functions [4]. Fc receptors that are part of the immunoglobulin G receptor family, FcγRs, bind to the hinge proximal region of the IgG C_H_2 domain with a 1:1 stoichiometry [15,16,17,18,19]. They are mainly found at the surface of immune cells and modulate the humoral and cellular immune responses [20]. The neonatal Fc receptor (FcRn) also binds to IgGs but plays a completely different role. The FcRn is found in hematopoietic and epithelial cells, where it modulates the half-life of IgGs by recycling them through transcytosis pathways [21]. Other Fc receptors, DC-SIGN and CD23, are part of the C-type lectin family and bind to the Fc domain at the interface between the two constant domains C_H_2/C_H_3 with a stoichiometry of two receptors for one antibody. The extent of their role in modulating IgG function is not yet fully understood but it is known that the DC-SIGN receptor triggers anti-inflammatory pathways [22]. In addition, complement-dependent cytotoxicity (CDC) is also activated by the binding of the complement component C1q to the Fc of multiple antibodies opsonizing a pathogen [23]. The binding of all Fc receptors is influenced by the type of glycan present at two conserved glycosylation sites, i.e., each asparagine residue (N297) of the Fc heavy chains. The Fc glycans are majorly biantennary with a heptasaccharide core. The Fab and Fc domains are connected by a flexible unstructured hinge region, resulting in a complex protein of about 150 kDa [24].

Several modifications can occur during the production of mAbs. They include oxidation, deamidation, enzymatic cleavage, or, more importantly, variation in the IgG glycosylation profile [25]. Their effects upon binding to FcγRs have been examined (Table 1), which revealed that asparagine deamidation reduced binding to the low-affinity FcγRs, i.e., FcγRIIa/b and FcγRIIIa/b, while deglycosylation strongly decreased or completely inhibited binding to all FcγRs [26,27].

The effects of different types of glycans, however, are more diverse and subtle due to a wide range of possible glycan chains present at N297. At the molecular level, the key role of the Fc N-glycans is to stabilize the Fc moiety of the IgGs in an open horseshoe-like conformation that increases the IgG interactions with the various FcγRs [28]. One branch of the biantennary glycans, the α1,6 arm, interacts with the protein backbone of the C_H_2 domain while the other arm, the α1,3 arm, is mobile in the space between the two C_H_2 domains and interacts with the opposite N-glycan [29]. The length and composition of both arms influence flexibility of the C_H_2 domains and the level of openness of the Fc moiety [30]. This modulation of the Fc flexibility, in turn, impacts the binding affinity of the Fc domain to the FcγRs.

### 2.2. FcγR Family

The Fcγ receptor family comprises three classes of receptors (Table 2): FcγRI, FcγRIIa/b/c, and FcγRIIIa/b. Together, these receptors play an essential role in the humoral and cellular immune responses by engaging with immune complexes. FcγRs are found on the surface of immune cells and modulate their responses such as phagocytosis, ADCC, antigen presentation, and antibody production [20]. Signalling occurs through either an immunotyrosine-like activation motif (ITAM) for activating receptors (FcγRI, FcγRIIa/c, FcγRIIIa) or an immunotyrosine-like inhibitory motif (ITIM) for the only inhibitory receptor, FcγRIIb [31]. Each receptor engages the Fc region of IgGs with a distinct affinity which is further influenced by key polymorphisms across the receptor family. Notably, the FcγRIIa can be expressed with arginine (R) or histidine (H) at position 131, with the arginine increasing its affinity for IgGs. The FcγRIIIa also presents a polymorphism at position 158, where a valine (V) or phenylalanine (F) can be found. The FcγRIIIa_V158_ has a significantly higher affinity for the IgGs than its counterpart [32]. Because of their impact on the FcγR-IgG interactions, it is important to consider these polymorphisms in the design (and administration) of therapeutic mAbs to patients.

The FcγRI structurally distinguishes itself from the other Fcγ receptors by its third extracellular domain. It also has the highest affinity for IgGs with a thermodynamic dissociation constant $K_{D}$ between 10^−8^ M and 10^−9^ M [27,33,34]. The role of its third domain is still debated, but a recent study suggested that it could act as a spacer enabling a more pronounced change in the Fc conformation upon binding, hence contributing to its higher affinity for IgGs [35]. The Fc binding site of all FcγRs, FcγRI included, has, however, been identified on the second domain where an FG loop interacts with the Fc C_H_2 domains [18,36]. However, the FG loop of the FcγRI is characterized by a positively charged KHR motif and a shorter length than the corresponding FG loop in the other receptors, which enables close contact with the Fc domains. Changing the KHR motif for neutral or negatively charged amino acids results in a 2- to 30-fold decrease in affinity. Introducing the FcγRI FG loop in FcγRIIIa increases its affinity for IgGs by 15-fold [18]. Kiyoshi and colleagues also reported that a unique hydrophobic pocket at the binding site of FcγRI may also increase its affinity for IgGs by entrapping the Fc L235 [35].

The higher affinity enables the FcγRI to bind monomeric antibodies, and therefore, it is suspected that this receptor is almost always in interaction with an IgG [37]. However, it is possible to observe differences for the IgG isoforms, with a greater affinity for IgG1 and IgG3 followed by IgG4 and finally IgG2 [10]. FcγRI is expressed on most immune cells such as monocytes, macrophages, and dendritic cells but also neutrophils, mastocytes, and eosinophils [38]. Its role in modulating immune responses is still unclear, but a recent study has revealed that it is involved in the production of reactive oxygen species (ROS) by neutrophils. Incubation of neutrophils with aggregated-IgGs causes degranulation and increases the surface expression of FcγRI following FcγRIIa engagement [39].

Three distinct isoforms of FcγRII are known: FcγRIIa, FcγRIIb, and FcγRIIc. These receptors are about 40 kDa and are present in the largest variety of human immune cells [34,36]. However, their affinity is the lowest, with a $K_{D}$ of about 10^−6^ M [13]. The FcγRIIb is mostly expressed on basophils, mastocytes, monocytes, macrophages, and dendritic cells, and it is the only inhibitory receptor reported so far, thus defining the activation threshold of the immune responses. FcγRIIa/c transduce activation signals in macrophages, monocytes, dendritic cells, and NK cells [22,39]. The binding mechanism of FcγRII to Fc has been debated, with early studies suggesting a 2:1 receptor:IgG complex [40]. However, new observations based on isothermal microcalorimetry (ITC) and NMR experiments indicated a 1:1 binding stoichiometry, further supported by crystallographic studies presenting the Fc moiety in interaction with only one FcγRIIa [36,41]. The FcγRIIa binds to the Fc lower hinge region in an asymmetrical way which enables contact with one of the Fc N-glycan [36].

Finally, the FcγRIII (or CD16) is expressed as two isoforms: FcγRIIIa and FcγRIIIb. Both isoforms bind IgGs with an affinity comprised between 10^−5^ and 10^−7^ M and with a 1:1 stoichiometry [11,13]. The FcγRIIIb is unique to neutrophiles and has a GPI anchor as the transmembrane domain. It is still unclear how this receptor transduces the signal to the cell. One hypothesis is that FcγRIIIb associates with FcγRIIa to activate degranulation and phagocytosis [38]. Multiple studies have also reported that FcγRIIIb can induce Ca^2+^ mobilization, nuclear factor activation, or neutrophiles extracellular traps (NETs) [38]. Compared to FcγRIIIb and other receptors, the impact of the FcγRIIIa polymorphism at position 158 (V/F) upon IgG binding is significant; it may contribute to better control of the immune responses triggered by FcγRIIIa [11,39]. In fact, this receptor is primarily expressed at the surface of natural killer (NK) cells and is responsible for initiating ADCC, an efficient immune response enhancing the effector functions of therapeutic mAbs [32]. High-resolution models show a similar asymmetric binding mechanism as for FcγRII, which involves one N297 glycan. Recent approaches enhanced the asymmetry of the complex by inducing specific mutations in the Fc region, which translated into higher affinity with FcγRs, in particular with FcγRIIIa [42]. Other than protein-protein interactions, the N-glycosylation of both the Fc and the FcγRIIIa impacts their interactions. There are five glycosylation sites on FcγRIIIa, and two of them enhance (N162) or decrease (N45) its affinity for IgG. The carbohydrate-carbohydrate interaction between the receptor N162-linked glycan and the Fc N297-linked glycan is favoured by an afucosylated Fc glycan [43]. On the other hand, the removal of the receptor N45-linked glycan resulted in a higher affinity for IgG. It was suggested that the presence of the N45 glycan induces steric hindrance between the N45 glycan and one Fc heavy chain blocking the Fc-FcγRIIIa interaction [44].

### 2.3. Impact of Glycosylation

One trait of the Fc N-glycosylation that stands out in several studies is the effect of core fucosylation [43,45]. A lack of core fucose has been directly associated with a significant increase in the binding affinity to FcγRIIIa [13,46]. The absence of fucose is suggested to enable glycan-glycan interaction between the glycan on N297 of the Fc region of IgG and the glycan on N162 of FcγRIIIa [43,45,47]. Based on those findings, afucosylation methods are now incorporated into the development of mAbs with improved capacity to mediate ADCC [9].

Other than fucose, the presence of a bisecting N-acetyl glucosamine (GlcNAc) and high-mannose glycans have also been associated, to a lesser degree, with enhanced ADCC activation via FcγRIIIa binding [48,49]. However, many studies have suggested that this enhanced ADCC activation might be only due to the fact that these glycan subtypes have a lesser core fucosylation level. Both high-mannose and bisecting GlcNAc N-glycans are also known to increase IgG affinity for the mannose-binding lectin (MBL), which initiates the lectin complement pathway cascade via the opsonization of the target cells [50]. Compared to complex N-glycan types, the high-mannose N-glycans have been shown to induce faster clearance of IgGs: it was observed that after administration of therapeutic IgG1 to patients, the high-mannose glycoforms were cleared more rapidly, compared to glycoforms with terminal galactose or bisecting GlcNAc, which levels remained constant for 34 days [51]. The IgG N-glycans are generally of the complex type with two arms ending with monosaccharides that are either galactose or sialic acid (Neu5Ac) [41]. Several studies have linked sialylation with a longer IgG serum half-life [52]. A recent study linked this effect with an increased binding to FcRn [52]. Terminal sialylation is also often associated with anti-inflammatory properties. Several research groups have correlated this effect to a reduced affinity for FcγRIIIa; others associated sialylation to increased conformation flexibility of the IgG Fc, which would in turn favour binding to DC-SIGN [14,53]. However, the effects of sialylation are still debated, with others showing that α2,6-sialylation has no significant influence on the IgG-FcγRIIIa interactions (compared to terminal galactosylation) and that the observed anti-inflammatory activity was independent of sialylation [46,54,55,56,57]. On the other hand, terminal galactosylation increases the affinity between IgG and FcγRIIIa [14,27,46,54,56].

Research on therapeutic mAbs’ quality attributes is increasing and influences the development of the next-generation antibody-related products and biosimilars [57]. Glycosylation may represent the most crucial challenge for biosimilar mAb development because it can influence the binding, immunogenicity, and effector activity of a mAb through its interactions with the various Fc receptors, specifically the FcγRs for IgGs. Recent investigations lean toward a better understanding of the IgG-FcγR binding mechanisms, offering new opportunities to enhance therapeutic mAbs [33,43]. This has been achieved thanks to kinetic studies relying on SPR biosensors. However, the recorded kinetic profiles for IgG-FcγR interactions have been acknowledged to be complex, i.e., they deviate from the 1:1 Langmuir kinetic model, despite biophysical studies indicating a 1:1 IgG-FcγR stoichiometry. The next section summarizes the evolution of the SPR-based studies on IgG-FcγR interactions to underline the consensus and discrepancies across their affinity assessment.

## 3. Surface Plasmon Resonance Biosensing

**Figure 1:**
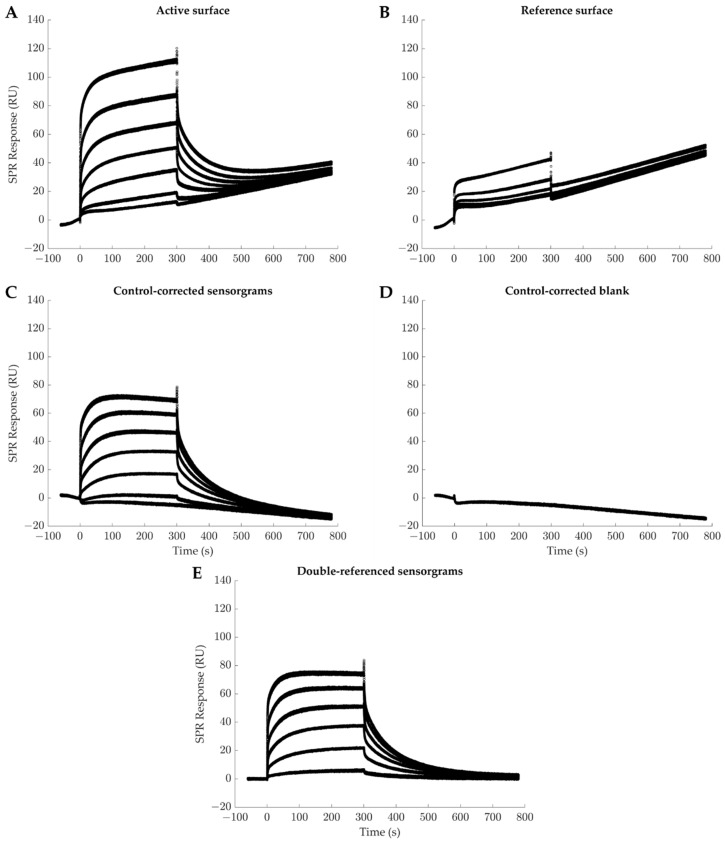
Trastuzumab (analyte) binding to FcγRIIIa_V158_ (ligand) recorded with a Biacore T100 instrument. Triplicate injections of six concentrations of Trastuzumab (20, 100, 250, 500, 1000, and 2000 nM) and buffer injection as blank were performed for 300 s, followed by 480 s of running buffer injection. The experiment is run over two surfaces: (**A**) an experimental surface presenting 20 RU of FcγRIIIa_V158_ immobilized and (**B**) a mock surface. (**C**) Control-corrected sensorgram resulting from the subtraction of data measured on the mock surface from the experimental surface data. The control-corrected data of the blank (**D**) is then subtracted from the experimental control-corrected data resulting in a double-referenced sensorgram (**E**).

Over the past two decades, a growing body of literature has reported the use of surface plasmon resonance (SPR)-based biosensors to analyze the IgG-FcγR interactions. For a detailed description of the SPR phenomenon and the commercialized SPR biosensors, the reader is referred to other reviews [58,59]. These instruments allow for real-time and label-free measurements of the interactions between a ligand (immobilized at the biosensor surface) and its injected biological partner, referred to as the analyte. Since data is collected in real time, it is possible to extract both kinetic ($k_{a}$ and $k_{d}$) and thermodynamic ($K_{D}$) constants that characterize the interactions under study [58]. As among all SPR biosensors, the Biacore instruments (now commercialized by Cytiva, formerly GE Healthcare) remain those prevalently used for the study of the IgG-FcγR interactions, we will thus now describe the steps of most Biacore experiments. With these instruments, one cycle, called a sensorgram, can generally be divided into three phases corresponding to various injections over a surface on which the ligand has been immobilized. These phases are (i) a buffer injection phase (to define the baseline), (ii) an analyte injection phase (often referred to as the association phase), followed by (iii) another buffer injection phase during which the ligand-analyte complexes dissociate (the dissociation phase). The temperature, the flowrate, the analyte concentration, and the injection durations can be adjusted for each cycle. To ease subsequent data analysis, it is critical that the original level of free and biologically active ligand remains constant from one sensorgram to the other. That is, either all the analyte molecules are completely removed (the signal is then back to the initial baseline level if the ligand is covalently bound to the surface) or the analyte-ligand complexes are detached from the surface prior to recapturing the same amount of fresh ligand molecule (if the ligand is non-covalently captured at the biosensor surface) [60]. A regeneration step is often used to reach this goal. To take into account non-specific interactions that may occur between the ligand and the biosensor matrix as well as to eliminate artifacts due to the operation of the biosensor (e.g., differences in refractive indexes when switching from one solution to another, electric perturbations due to the biosensor’s moving parts, etc.), Myszka has proposed a robust operating protocol, the double referencing approach, now adopted by most Biacore users and demonstrated in Figure 1 [61]. This corresponds to i) subtracting the signal recorded from the same sequence of injections performed over a reference surface (i.e., without any ligand) and ii) subtracting to this control-corrected sensorgram a control-corrected sensorgram corresponding to the same sequence of injections performed with buffer rather than the analyte (i.e., [analyte] = 0 M) [61].

The kinetics may then be derived by globally fitting a data set of double-referenced sensorgrams collected for various concentrations of analytes to a mathematical model describing the interaction [62]. As the analyte and buffer injections are performed in a continuous fashion, the concentration of the sample in the flow cell is constant in time. This simplifies the modelling of the sensorgrams. When the binding kinetic is simple, the analyte injection phase contains information about the association (k) and dissociation kinetic constants ($k_{d}$), while the dissociation phase contains information about the dissociation constant ($k_{d}$) only [58]. It is then possible to calculate the thermodynamic dissociation constant $K_{D}$ equal to the $k_{d}/k_{a}$ ratio. Note that this thermodynamic constant can also be deduced from the plateau values of the various sensorgrams if an equilibrium is reached during the analyte injection phases.

The choice of the ligand immobilization method, ligand density, flowrate, and buffer composition may affect the recorded data and thus bias their interpretation. Therefore, the experimental conditions must be optimized first to eliminate or at least minimize potential artifacts, hence facilitating subsequent data analysis: a kinetic model needs to be selected with care, keeping in mind that multiple factors, other than the biological interaction itself, can have an impact on the fit [63]. Generally, the simplest model describing the studied interaction leads to more reliable kinetics parameters. However, in the case of IgG-FcγR interactions, deviations from a 1:1 Langmuir kinetic model have been acknowledged even though the interactions are known to follow a 1:1 stoichiometry. The inability to depict the IgG-FcγR interactions with a simple Langmuir model is a common thread in studies over the past 20 years and has translated into a large range of reported $K_{D}$ values. It is thus pertinent to wonder whether the apparent complexity of the SPR data sets for IgG-FcγR interactions comes from ill-defined experiments or from a complex biological situation.

### SPR-Based Analysis of the IgG- FcγR Interactions

**Figure 2:**
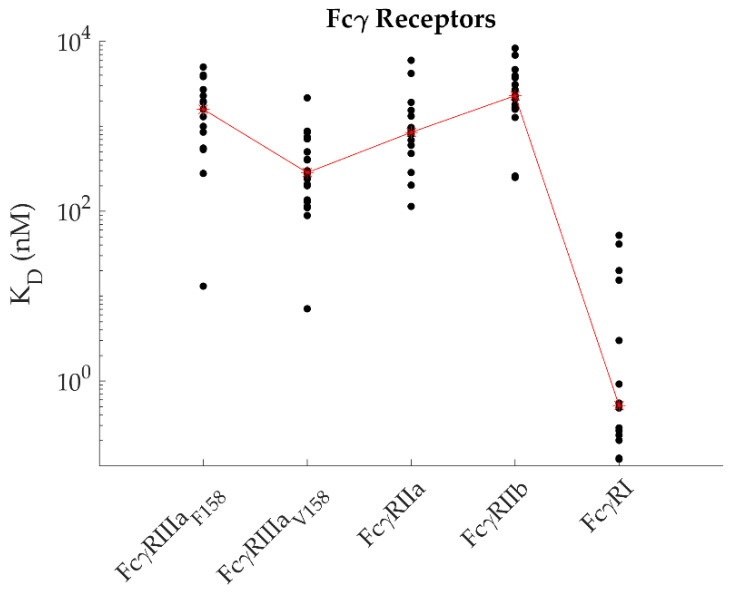
Affinities reported in the literature between FcγRs and IgGs (35 studies). The red line crosses the median point values specific to each receptor.

SPR studies have mainly focused on five FcγRs: FcγRI, FcγRIIa, FcγRIIb, FcγRIIIa_V158,_ and FcγRIIIa_F158_, because of their wide distribution across the immune cells and the immune responses that they trigger. Figure 2 presents an overview of the apparent $K_{D}$ values that have been reported for these receptors binding to IgG1. As a reference, the median specific to each receptor is marked in red. The results are also presented in Table A1 of Appendix A, along with some of the main parameters of the SPR experiments, i.e., the choice of ligand, the immobilization strategy, and the analysis model. In many cases, the apparent $K_{D}$ values for a given receptor vary widely, even when the same experimental approach has been adopted. For example, for amine immobilized IgG1 binding to soluble FcγRI, differences in $K_{D}$ of almost 200 folds were reported (Table A1 Appendix A). However, it is important to keep in mind that several other experimental parameters than those analyzed in the next Figures and not always reported in the original publication might have varied from one SPR campaign to another and could have impacted the results. These include the flow rate, ligand density, regeneration protocol, range of analyte concentrations, and injection duration. The source of the proteins under study (especially the cell platform used to express them) also differs from one study to the other. Since post-translational modifications impact the IgG-FcγR interactions, it is also important to consider the production and purification processes when comparing these SPR data.

Despite the great diversity of receptor origins and SPR protocols, general trends have emerged in the literature:

First, several research groups [10,13,46] compared various FcγRs within the same study. Their results thus provide at least a semi-quantitative (if not fully quantitative) idea of the affinity differences between these receptors as the SPR methodology was consistent in each of these studies. The IgG-FcγR interactions are ranked, from the highest to the lowest affinity, as follows: FcγRI > FcγRIIIa _V158_ > FcγRIIIa_F158_ > FcγRIIa > FcγRIIb.

**Figure 3:**
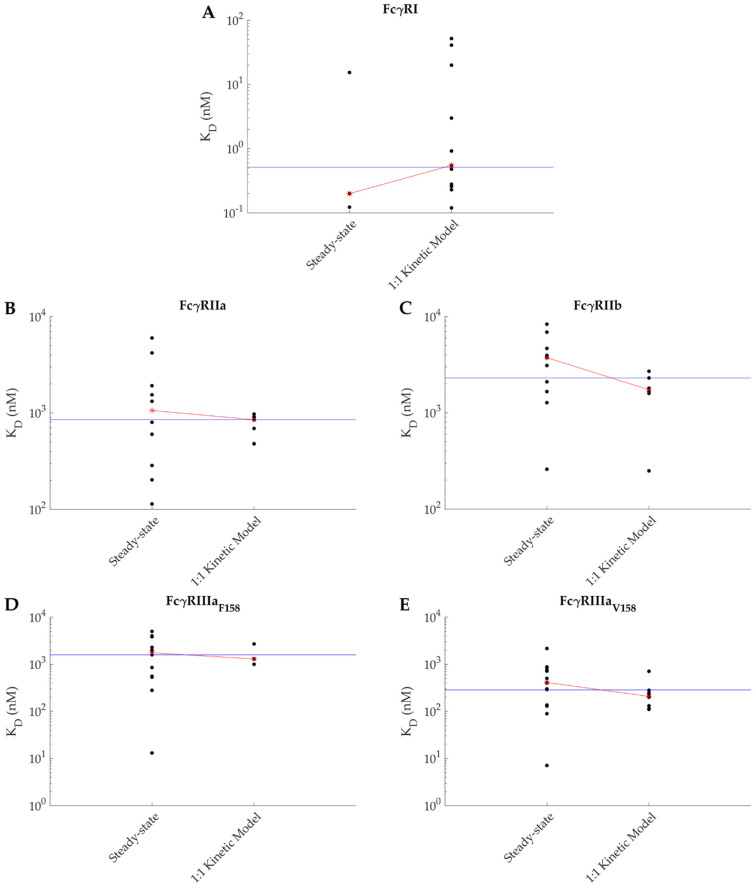
Affinities reported in the literature between IgG and (**A**) FcgRI, (**B**) FcgRIIa, (**C**) FcgRIIb, (**D**) FcgRIIIa_F158,_ and (**E**) FcgRIIIa_V158_ depending on the analysis model (steady-state or 1:1 Langmuir kinetic model). The blue line represents the median of all values combined, while the red line crosses the median point values specific to each analysis model.

Second, most of the apparent $K_{D}$s were determined from equilibrium values (from the plateau values of the sensorgrams) rather than from the ratio of the kinetic constants. This once again reflects the inability to fit the SPR kinetic data with a simple kinetic model. This is the approach of choice for FcγRIIa/b as they both have a fast association and dissociation kinetics when interacting with IgGs. While these fast kinetics are difficult to evaluate precisely due to the sampling frequency of current instruments (10 Hz) and due to a very fast transition phase (about a second) and potential mass transport constraints, these kinetics, however, allow to rapidly reach steady state. Many studies have examined both FcγRII interactions, and even if the range of reported $K_{D}$ across these studies is wide (Figure 2 and Table A1, Appendix A), a consensus is clear: FcγRIIa has a higher affinity than FcγRIIb. Figure 3 presents the data points reported in the literature according to the analysis model used for all receptors. The median of all data points combined has been identified by a blue line. Note that the IgG-FcγRI is an exception to the steady-state analysis trend, more likely due to the fact that the dissociation rate of the IgG-FcγRI complex is too slow to easily reach equilibrium. However, one study by Bruhns et al. (Table A1, Appendix A) did analyze the IgG-FcγRI interactions with a steady-state approach (amine coupled receptor and polyclonal IgGs as analyte) [10]. Since polyclonal IgGs were used, complex kinetics were expected, hence the choice of a steady-state approach [10]. Of interest, the apparent $K_{D}$ value (around 15 nM) was found very similar to those deduced from kinetic analyses with the same immobilization strategy (Table A1, Appendix A).

**Figure 4:**
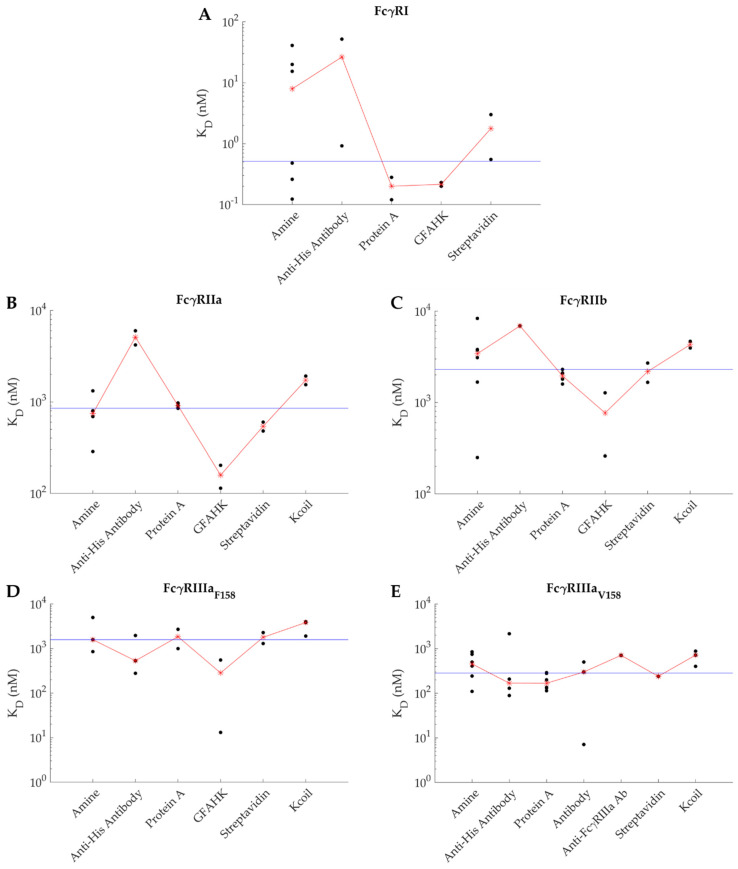
Affinities reported in the literature between IgG and (**A**) FcγRI, (**B**) FcγRIIa, (**C**) FcγRIIb, (**D**) FcγRIIIa_F158_ and (**E**) FcγRIIIa_V158_ depending on the immobilization strategy. GFAHK stands for Goat F(ab′)2 anti-human kappa antibody. The blue line represents the median of all values combined, while the red line crosses the median point values specific to each immobilization strategy.

Third, the amine coupling approach is the most widely used immobilization protocol, as it is the simplest to apply (Figure 4 and Table A1, Appendix A). It takes advantage of the available amine groups on the N-terminus and side chains of the lysine residues of the protein to be immobilized. Thus, this strategy must be applied with caution since, depending on the number of lysine residues and the reactivity of their sidechain, the protein may well be immobilized heterogeneously, which in turn, may translate into heterogeneous (more complex) kinetics. That is, some amine group participating in the coupling may be close to the ligand-binding site, thus impacting its interaction with the analyte. Another important aspect related to covalent coupling is that the regeneration of the surface must be carefully handled since the bioactivity of the ligand must be preserved from one sensorgram to the other for a valid kinetic and thermodynamic analysis. This is especially important for the FcγRI since its dissociation for IgGs never reaches completion on the time scale of an SPR experiment. Adequacy of the regeneration procedure must be tested by performing multiple injections of the analyte at the same concentration. Altogether, the use of amine coupling may not be the best method for an in-depth characterization of the IgG-FcγR interactions. Interestingly, Lu et al. performed experiments with FcγRI as the ligand or analyte (amine coupling) [64]. The apparent $K_{D}$s were of 20 nM and 40 nM when the IgG1 was used as the ligand or the analyte, respectively. These values are close, considering the potential bias amine coupling might have introduced; their similarity also confirms the 1:1 stoichiometry of the interaction.

The caveats related to covalent immobilization could be overcome by capturing the ligand via a capture agent (previously covalently immobilized) on the biosensor surface [58]. Despite the increase in the amount of ligand needed (since fresh ligand has to be captured for each sensorgram), this strategy is more and more adopted by SPR users due to its numerous benefits: (i) the tags exploited for protein purification, e.g., the histidine-tag can also be leveraged in SPR experiments with a specific capture agent (Ni-NTA or anti-His tag antibodies); (ii) the same biosensor surface may capture different amounts of the same ligand or different ligands, easing experiment standardization and subsequent data comparison; (iii) surface regeneration is facilitated since the analyte and the ligand are removed at the end of each cycle (ligand bioactivity is warranted from one sensorgram to the other).

Several research groups have reported the use of a capture agent for the study of the interactions between IgGs and FcγRs, as presented in Figure 4. Among them, several capture agents directly leverage regions on the IgGs, e.g., the goat F(ab′)2 anti-human kappa antibody or the Protein A, while others are tag-specific, such as the anti-his antibody, the streptavidin, or the Kcoil peptide. The approach relying on streptavidin has been commercialized as a ready-to-use kit by the Biacore^TM^ manufacturer. This kit (the Biotin CAPture kit) relies on a biosensor chip displaying a single strand of DNA. The experimental design consists of capturing streptavidin coupled to the complementary DNA strand prior to capturing the biotinylated ligand via its interaction with surface-captured streptavidin [65]. Surface regeneration is achieved by promoting the dissociation of the DNA strands. Despite its versatility, this strategy is less reproducible in the amounts of captured ligand than others [13], most likely due to the additional step corresponding to the capture of streptavidin prior to that of ligand. Our laboratory has also developed an alternative strategy based on de novo designed interacting peptides, the E/K coils, known to form a specific and stable coiled-coil complex [66]. In our approach, the ligand is produced with a 35-amino acid-long peptidic tag (the Ecoil), allowing for its capture on a biosensor surface displaying its partner Kcoil peptide [13].

Several studies applied an oriented immobilization method to analyze the IgG-FcγR interactions. However, caution must prevail in the choice of these methods as well. Indeed, two studies revealed potential biases from the use of antibodies or streptavidin as capture agents [13,27]. In particular, the use of anti-histidine antibodies to capture tagged receptors was shown to induce a characteristic artifact where the signal reaches a steady state then decreases over time instead of staying stable [27,67]. It was suggested that this artifact could come from interactions between the FcγRs and the Fc region of the anti-histidine antibodies. If true, this hypothesis may also raise concerns for any other capture antibody such as the goat F(ab′)2 anti-human kappa antibody that had been used as a capture agent (Figure 4). Protein A or streptavidin are suitable alternatives to capture antibodies, but there are still concerns about the streptavidin-biotin strategy. In fact, because streptavidin is tetrameric, one may wonder if this experimental approach increases the chances of steric hindrance and rebinding artifacts as more than one ligand can be captured by the same streptavidin molecule. The use of Protein A overcomes these potential artifacts with a well-defined 1:1 stoichiometry for IgG. It has also been demonstrated that the use of the Kcoil/Ecoil peptidic system enables a stable and oriented capture of the FcγRs tagged with the Ecoil peptide. Of interest, the performances of this capture approach for the specific study of the IgG-FcγR interactions was compared to other experimental protocols relying on an anti-His antibody or streptavidin-biotin interactions. It was demonstrated to increase the biosensor surface lifetime as well as the reproducibility of the SPR assay [13].

Other than the capture strategy, the experimental injection parameters, especially the flow rate, can also have an impact on the result. In early studies, low analyte injection flow rates (10 μL/min or lower) were common [40,68]. Despite the increase in material consumption, it is highly recommended to use faster flow rates (>30 μL/min) to reduce mass transport limitation. This artifact is one of the most common problems in SPR-based experiments: it occurs when the binding rate of the analyte to the ligand is faster than the diffusion of the analyte from the bulk to the biosensor surface [69]. It can therefore lead to apparent kinetics that are not an accurate depiction of the interaction under study. Another way to reduce mass transport limitation is to minimize the quantity of captured ligand to its minimum while maintaining an acceptable signal-to-noise ratio. This practice also minimizes the risks of aggregation, steric hindrance, and rebinding artifacts [69]. Further work on a better understanding of the transport in protein-polymer thin films and the development of nanopore arrays are also potential avenues to help overcome mass transport limitation artifacts [70,71,72,73]. In fact, the use of nanopore arrays has been shown to enhance transport and sensitivity, thus becoming a growing niche within SPR-based biosensing novel methods [70,71,73].

It is clear that the parameters of an SPR experiment influence the outcome and might hinder the identification of simple kinetics. The variety of SPR-based experimental designs applied to the case of the IgG-FcγR interactions from 2001 to 2020 could thus explain, in part, the wide range of reported $K_{D}$. However, there is also much variety in the production processes of IgGs and FcγRs in those studies. Some researchers used a *Pichia* strain modified to produce human glycans, while most of the latest studies have turned to human embryonic kidney 293 (HEK293) or to Chinese hamster ovary (CHO) cell lines [13,74,75,76]. In fact, these two cell lines are widely used in the biopharmaceutical industry. They are well characterized, and several experimental strategies have been developed to influence the output protein glycoforms. These were applied to influence the N-glycosylation of IgGs and test its impact on FcγR binding by SPR, particularly for the IgG-FcγRIIIa interactions. There is a variety of strategies to influence glycosylation that were used across studies and that led to different levels of IgG glycoforms. While some of these methods are presented in the next section, a growing number of studies now take this factor into account by producing IgGs enriched in a particular glycoform.

In parallel to the exploration of the impact of IgG glycosylation, the latest studies have highlighted an additional complexity by taking into consideration FcγR glycosylation. Until now, noticeable impacts have mostly been observed for both FcγRIIIa_F158_ and FcγRIIIa_V158_ only. Several groups have focused on the impact of FcγRIIIa glycans by removing glycosylation sites from this receptor (hence reducing receptors’ macro-heterogeneity) [12,33,76]. The impact of the N-glycan at position N162 of the FcγRIIIa is consensual since its absence leads to a decrease in its affinity for afucosylated IgGs by at least one order of magnitude. The molecular mechanisms explaining the role of the FcγRIIIa N162 glycan were suggested to rely on its glycan-glycan interactions with the Fc glycan, and that the absence of fucose on the IgG would reduce steric hindrance [43,47]. The N-glycan at position N45 of FcγRIIIa has also been shown to have an impact on the IgG-FcγRIIIa interactions. Its removal led to a higher affinity for afucosylated IgGs [76]. The other three glycans at positions N38, N74, and N169 of the FcγRIIIa showed no influence on the receptor interactions with IgGs. Other than removing each N-glycan of FcγRIIIa, it was also shown that the type of glycans present at these FcγRIIIa glycosylation sites have an influence on the IgG-FcγRIIIa interactions (this is referred to as micro-heterogeneity): FcγRIIIa presenting high-mannose N-glycans has a higher affinity for the IgGs [77]. Hayes and colleagues also concluded that tetra-antennary glycans and sialylated glycans obtained with CHO cells negatively influenced binding to IgGs [67].

## 4. Toward a Better Understanding of the IgG-FcγR Interaction

As the therapeutic mAbs market is growing, modulating the IgG-FcγR interactions to enhance the IgG therapeutic efficacy and developing robust assays to test the critical quality attributes of these therapeutic IgGs routinely are of prime importance. SPR-based biosensing has played a significant role in both aspects, but the intrinsic complexity of the role of glycans upon the IgG-FcγR interactions has impeded their in-depth kinetic characterization. In this section, we review some of the latest methods to achieve a higher glycoform homogeneity of IgG lots. We also explore different numerical approaches to enhance the analysis of SPR data presenting complex kinetics such as the IgG-FcγR interactions.

### 4.1. Glycosylation Control

Regarding fucosylation, the use of fucosyltransferase 8 (FUT8) inhibitors such as the 2-F-peracetyl fucose [78] and the 2-deoxy-2-fluoro-L-fucose [14,46,49,79] has been tested and significantly reduced the rate of fucosylation. Of interest, the addition of 2-F-peracetyl fucose to the feeding media led to 90% of afucosylated IgGs produced in CHO cells [78]. Comparatively, the addition of 2-deoxy-2-fluoro-L-fucose was shown to result in 85% of afucosylated IgGs in HEK293 cells [49]. Another common method to reduce fucosylation relies on transfecting cells with the cDNA encoding the bacterial GDP-6-deoxy-D-lyxo4-hexulose reductase (RMD) to block the production of fucose. This enzyme converts GDP-4-keto-6-deoxy-D-mannose to GDP-D-rhamnose, leading to negative feedback on GMD activity and consequently to a low GDP-fucose level [80]. The expression of RMD during the production of IgG1 has been reported to result in levels of afucosylated IgGs ranging from 73% [49] to 98% [80]. Another approach relying on the expression of the N-acetylglucosaminyltransferase III (GnTIII), an enzyme catalyzing the addition of a bisecting GlcNAc onto the central β-mannose of the glycan [12], has also been explored with reported levels of afucosylation up to 90% [12]. Here, the addition of a bisecting GlcNAc indirectly decreases fucosylation by blocking the action of α1,6-fucosyltransferase during the IgG biosynthesis. The overexpression of the GDP-6-deoxy-D-talose synthase (GTS), an enzyme that converts GDP-4-keto-6-deoxymannose to GDP-6-deoxy-D-talose, was recently shown to result in more than 80% afucosylation levels in CHO cells [81]. Using gene-editing methods, different enzymes involved in the GDP-fucose biosynthesis pathway were also targeted. Cell lines no longer encoding α1,6-fucosyltransferase (FUT8 knockout) were generated, leading to the complete suppression of fucosylation [82]. Other groups investigated the generation of an FX knockout (GDP-4-keto-6-D-deoxymannose epimerase/GDP-4-keto-6-L-galactose reductase knockout) CHO cell line that enables control over the level of desired fucosylated or afucosylated antibodies by varying the addition of fucose to the cell culture media [83].

Besides fucosylation, the modulation of galactosylation and sialylation has also been studied. For example, supplementation of culture media with different additives such as a combination of cytidine, fucose, and uridine, or manganese chloride and galactose were shown to increase galactosylation up to 50% [78]. In addition, the co-expression of the β1,4-galactosyltransferase 1 or β1,4-galactosyltransferase 2 increased the level of galactosylation up to 70% and 50%, respectively [49]. Combining the expression of ST6Gal1 and galactosyltransferase resulted in 44% of sialylated IgGs [49]. It was also observed that the co-expression of α2,6-sialyltransferase (ST6Gal1) and β1,4-galactosyltransferase 1 (β4GT1) in a CHO line naturally expressing only α2,3-sialyltransferase was necessary to achieve α2,6-sialylation-enriched glycosylation profiles [84]. Another interesting approach to modulate the galactosylation and sialylation of IgGs is to mutate the Fc region to increase the accessibility of its glycosylation site to galactosyltransferase and sialyltransferase [85]. This strategy, combined with the overexpression of sialyltransferase, enabled the production of IgGs with 77% of α2,6-sialylation [85]. On the other end, to reduce the level of galactosylation, the galactose analog 2-deoxy-2-fluoro-d-galactose (2FG) has been identified as a specific blocker of galactosylation [49]. Its addition resulted in 91% of agalactosylated IgGs. To decrease sialylation, CHO and NS0 cell lines secreting both IgG and sialidase A were developed to remove sialic acid during IgG production [86].

In vitro remodelling of IgG glycans is also commonly reported. Several groups increased the galactosylation of IgGs with an enzymatic treatment relying on the β4GT1 and the UDP-galactose as a substrate [46,54,87]. Sialylation level was also modulated with a sialyltransferase (ST6GalI) and the precursor N-acetylneuraminic acid (CMP-NANA). The proportion of galactosylation or sialylation can be further controlled by varying the enzyme and substrate concentrations as well as the incubation time [49,54]. It is also possible to remove terminal sialic acid and galactose with sialidases and β-1,4 galactosidases mixed sequentially with the IgGs [54,87]. Finally, the use of endoglycosidases being specific to the whole glycan tree rather than only one residue was also explored. In this approach, an endoglycosidase is able to remove the N-glycans of a protein while another endoglycosidase transfers a new already-assembled glycan [53,55,56,88]. However, these in vitro engineering approaches remain expensive, long, and poorly scalable as of today [54]. Moreover, they only provide enrichment of a specific glycoform [46].

Finally, mathematical models based on kinetic metabolic reaction and carbon sources influencing the glycosylation processes are also being developed to optimize production conditions [89]: a number of kinetic metabolic reaction network models or metabolic flux analysis (MFA) models have been built to simulate the glycosylation process [90,91]. A newly developed approach, the glycan residues balance analysis or GReBA, was proposed to link the concentration of sugars in the medium to the distribution of glycosylation patterns [92]. By simulating the synthesis of monosaccharides such as galactose, fucose, and sialic acid, and the glycosylation process, the authors were able to predict the glycosylation profile for steady-state cell cultures [92]. Furthermore, the same team designed a targeted feeding (TAFE) strategy for a perfusion cell culture to orient the cells toward a desired IgG glycosylation profile. Applying the TAFE strategy, different glycan profiles were obtained using different feeding regimes [93].

Altogether, this body of work opens the way to better predict and control glycosylation profile for IgG production. Combined with advances in SPR data treatment, these bioengineering strategies may further enhance our understanding of the role of glycosylation upon the modulation of the IgG-FcγR interactions.

### 4.2. SPR Data Analysis of the IgG-FcγR Interaction

SPR responses are typically modelled with a 1:1 Langmuir interaction between the analyte (A) and the ligand (L):

(1) $$A+L\begin{array}{c} \overset{k_{a}}{\to }\\ \underset{k_{d}}{\leftarrow } \end{array}AL$$

With $k_{a}$ and $k_{d}$ corresponding to the association and dissociation rate constants, respectively. The system is described by the following ordinary differential equation (ODE):

(2) $$\frac{dR}{dt}=k_{a}C_{TOT}\left(R_{max}-R\right)-k_{d}R$$

where $C_{TOT}$ is the concentration of analyte (being constant during the analyte injection phase and equal to 0 during the dissociation phase) and $R_{max}$ is the theoretical SPR response that would be obtained if an infinite concentration of analyte were injected. It is now acknowledged that such a model cannot properly fit the sensorgrams corresponding to the interaction between the various Fcγ receptors and the IgG1, whether the antibody (or its Fc region) is the ligand [46,77] or the analyte [11,13,33,76,94,95]. It is, however, possible to use the 1:1 Langmuir model to identify an apparent equilibrium constant ($K_{D}=k_{d}/k_{a}$ or $K_{A}=k_{a}/k_{d}$), as equilibrium plateau values ($R_{eq}$) can be reached during analyte injections for the FcγRIIs and FcγRIIIs, and they are well described by this model [11,13,33,46,76,77,95]. Complex interaction models also typically behave analogously to a 1:1 Langmuir model at equilibrium, as we will describe in the coming subsections.

By forcing Equation (2) to 0 (i.e., at equilibrium), we obtain:

(3) $$R_{eq}=\frac{R_{max}C_{TOT}}{K_{D}+C_{TOT}}$$

As mentioned earlier, for FcγRI binding to IgG, most research groups have used the 1:1 Langmuir model to identify kinetic parameters [13,33,46,94], mainly because the binding is too slow to reach equilibrium (and apply Equation (3)). Such an analysis leads to biased parameters as the model does not properly represent the data. This was depicted in many reports using different (optimized) SPR assays [11,13,33,46], and it is therefore unlikely that the poor fit to a simple 1:1 scheme is a result of a poorly designed assay.

All this highly suggests that IgGs interact with Fcγ receptors according to a more complex scheme(s). Many individual factors could explain the complexity of the interactions, and it is quite possible that a combination of these factors comes into play, even when experimental artifacts are discarded. The following section describes different models that could potentially be used to analyze the SPR sensorgrams and elucidate the FcγR-IgG interactions. For the identified parameters to have a physical meaning, the model must fit the data properly, but it is also necessary that it properly describes the interactions as they really occur at the biomolecular level. Hence, for each model, we will provide a biology-based rationale as to why the model could be of potential interest. One must, however, keep in mind that a more complex model means more parameters to identify and, therefore, a better fit to experimental data, whether or not the model is biologically pertinent.

#### 4.2.1. FcγR Glycosylation and Heterogeneous Ligand Models

Heterogeneity of the ligand population on the biosensor surface can cause a significant deviation from the ideal 1:1 Langmuir case. Such heterogeneity can hail from two sources mainly [96,97]. First, ligand molecules can be immobilized with varying orientations when a random (amine) coupling is used during the immobilization step. This artifact has already been discarded in several studies thanks to oriented capture protocols; in these studies, deviations from a simple 1:1 model were still observed. Hence, the complexity of the IgG-FcγR cannot be totally ascribed to this artifact.

Second, ligand heterogeneity may be intrinsic to the biological system since Fcγ receptors have multiple glycosylation sites that may be glycosylated in a heterogeneous fashion. Experimental results tend to confirm this assumption as receptor glycovariants have been reported to have different binding kinetics for the Fc region of IgGs [13,33,76,77]. This complexity may be reduced by deleting several receptor glycosylation sites [33,76,77], hence decreasing the variability of the system (i.e., macro-heterogeneity issues), but the remaining sites can still harbour distinct glycan chains. This micro-heterogeneity can, in turn, be reduced during or post-receptor production (see above).

Assuming a limited number of kinetically distinct glycovariants, this type of heterogeneity can be modelled by considering a homogeneous IgG (analyte, A) binding to multiple populations of ligand (FcγR, $L_{i}$) with distinct kinetic parameters $k_{a,i}$ and $k_{d,i}$:

(4) $$A+L_{i}\begin{array}{c} \begin{array}{c} k_{a,i}\\ \to \end{array}\\ \begin{array}{c} \leftarrow \\ k_{d,i} \end{array} \end{array}AL_{i}\;\;\forall \;i$$

This model can be translated to the following ODE system:

(5) $$\frac{dR_{i}}{dt}=k_{a,i}C_{TOT}\left(R_{max,i}-R_{i}\right)-k_{d,i}R_{i}\;\;\forall \;i\;R\left(t\right)=\underset{i}{\sum }R_{i}\left(t\right)$$

Here, each ligand population acts independently from the others, with no competition. Each ligand population has its own $R_{max,i}$, which corresponds to the SPR response that would theoretically be recorded if all ligand molecules of the i^th^ population had bound to an analyte molecule. Traditional analysis frameworks only enable the use of two classes of ligand [63,98], which is likely to be insufficient for the purpose of studying receptor-mAbs interactions due to the high number of distinct glycan chains which can be observed on FcγRs.

Svitel et al. [96] introduced a framework enabling the treatment of multiple populations of ligand. A mesh of affinities ($K_{A}$) and association kinetics ($k_{a}$) is constructed, and each node of this mesh represents a class of ligand. Fitting the model then comes down to identifying the contribution ($R_{max,i}$) of each node (or population of ligand) to the recorded sensorgrams. The SPR responses estimated by the model in this framework are obtained by summing the SPR responses of each node. This is obtained by integrating Equation (5) for all nodes. The fitting procedure suggested by Svitel et al. [97] comes down to adding units with $R_{max}=1$ to various nodes until the estimated responses correspond to the observed sensorgrams. One can represent the contributions of all the nodes on a heat map, for example. Nodes with a higher contribution are called peaks. A Tikhonov regularization scheme is used to penalize solutions with a large number of peaks and thus avoid overfitting.

Such a framework has been applied to various protein systems [96,97,99,100,101]. Notably, Svitel et al. [97] included the possibility to consider mass transfer limitations in the model, and Gorshkova et al. [101] suggested using prior knowledge of the distribution of ligand populations to obtain a narrower distribution around the main peak. As such, if secondary peaks appear, one can be more confident that they represent a significant contribution to the system, rather than simply measurement noise.

#### 4.2.2. Analysis of IgG N-Glycosylation Impact with More Sophisticated Models (N-Analytes)

As detailed above, enzymatic treatments performed post-production [13,33,102] or specific engineered cell lines [103] permit a more uniform glycosylation profile of a given IgG. However, obtaining a homogeneous solution of a single IgG glycoform does not seem achievable in the near term. Therefore, the observed deviations of the SPR response from the 1:1 Langmuir behaviour may come from the still-present heterogeneity in the glycosylation state of these glycan-engineered IgGs.

Si Mehand et al. [104,105] first demonstrated that it is possible to identify from the SPR recorded signal the kinetic parameters of two distinct analytes that are simultaneously injected over a given ligand. While the original intent of Si Mehand’s study was to increase the SPR biosensor throughput in a screening context, Gaudreault et al. [106] recently extended this theoretical framework to the case of N analytes. Here, the goal was to identify the kinetic parameters for each individual analyte within a complex analyte mixture, an asset if the analytes are difficult to separate from each other. Of interest, Gaudreault et al. demonstrated that the kinetic parameters extracted from mixtures of four analytes were similar to those identified with traditional single-analyte experiments. Such an approach is thus promising for the in-depth characterization of the various glycoforms mixtures of an IgG produced in mammalian cell cultures.

More precisely, the N glycoforms of the same IgG can be modelled as N analytes ($A_{i}$) competing for binding to a common Fcγ receptor (L) captured at the biosensor surface, each with their respective kinetic constants $k_{a,i}$ and $k_{d,i}$. This leads to N pseudo-reactions:

(6) $$A_{i}+L\begin{array}{c} \begin{array}{c} k_{a,i}\\ \to \end{array}\\ \begin{array}{c} \leftarrow \\ k_{d,i} \end{array} \end{array}A_{i}L\;\forall \;i=1,2,\ldots ,N$$

The model is described by the following ODE system, which can be integrated numerically:

(7) $$\frac{dR_{i}}{dt}=k_{a,i}F_{i}C_{TOT}R_{max,i}\left(1-\overset{N}{\underset{j=1}{\sum }}\frac{R_{j}}{R_{max,j}}\right)-k_{d,i}R_{i}\;\;R_{i}\left(0\right)=0,\;\forall \;i=1,\ldots ,N\;R_{TOT}=\overset{N}{\underset{i=1}{\sum }}R_{i}$$

where $F_{i}$ represents the set of fractions of each analyte in the mixture. The basis of the fitting method used by Gaudreault et al. [106] revolves around the fact that the model of Equation (7) exhibits the same behaviour at equilibrium as a 1:1 Langmuir model with the following parameters:

(8) $$K_{A,obs}=\overset{N}{\underset{i=1}{\sum }}F_{i}K_{A,i}=\frac{1}{K_{D,obs}}$$

(9) $$R_{max,obs}=\frac{\sum_{i=1}^{N}F_{i}K_{A,i}R_{max,i}}{K_{A,obs}}$$

Initial estimates of the parameters of the model are obtained via linear regressions and then used as starting points in an optimization routine. High molecular weight analytes, such as IgGs, usually have similar refractive index increments (RII), thus implying that the ratio of their respective $R_{max,i}$ should be proportional to the ratio of their molecular weights [63,104,107,108]. As different glycoforms of a unique antibody should exhibit negligible differences in their molecular weight, including only one $R_{max}$ common to every analyte is thus sufficient to analyze mixtures of IgG glycoforms.

Interestingly, Gaudreault et al. [106] also suggested a way to estimate the mixture composition (represented by the set of fractions $F_{i}$ of each analyte in the mixture), assuming that the kinetic parameters have been previously identified. This approach thus potentially paves the way to using SPR biosensing to determine the composition of IgG glycovariants mixtures if the glycovariants are characterized by distinct kinetics of interaction with their cognate receptors. One may indeed envision that mixtures of IgG glycovariants of known compositions (being first characterized by a traditional method such as HILIC) could be used to identify the kinetic parameters of each glycoform. Knowledge of these parameters could then be used to identify the composition of any mixtures of glycoforms of the same IgG.

So far, this framework has been applied and validated with a model system corresponding to the carbonic anhydrase II (as the ligand) interacting with several low molecular weight inhibitors of the enzyme (the analytes). The main limitation of the proposed approach is that N independent mixtures of the N analytes are required to identify their kinetic parameters. This could prove a limitation to the case of the IgG glycovariants, as a high number of distinct glycan chains can be observed on the Fc region of IgGs produced in mammalian cell culture. Pooling (kinetically) similar glycovariants as one analyte could partially solve this issue, although the question of which glycovariants to pool remains open.

#### 4.2.3. Rate Distribution Framework for N-Analyte Systems

An adaptative interaction distribution algorithm (AIDA) was developed by Zhang et al. [109] to identify the number of pseudo-reactions (N in Equation (6) or potentially the number of previously mentioned pools in the context of IgG-FcγR interactions) and identify the kinetic parameters describing those pseudo-reactions. The algorithm starts with constructing a graph of the natural logarithm of the recorded responses during the dissociation phase of the sensorgrams. If this graph is linear, then the sensorgrams can be properly explained by a single pseudo-reaction (N=1). If the graph is convex, then the data is indicative of the presence of more than one interaction (N≥2).

A rate constants distribution (RCD) is then constructed for each available sensorgram. A mesh of association ($k_{a}$) and dissociation ($k_{d}$) kinetic parameters are created, and the contribution of each node is identified using regularization methods, similar to what has previously been discussed in the case of multiple ligand populations. The rate distributions can be illustrated on heat maps. Counting the number of peaks on these heat maps gives the number of pseudo-reactions N observed in the data (number of kinetically different analytes necessary to explain the data).

A model with the identified N pseudo-reactions is then fitted independently for each sensorgram in the data set with peak values as starting points for an optimization routine. All the identified rates for all sensorgrams are then plotted on the same $k_{a}$ versus $k_{d}$ plot, and clustering methods are used to regroup these rates into N clusters, with each cluster corresponding to one pseudo-reaction. Middle points (medians) of these clusters correspond to the identified $k_{a,i}$ and $k_{d,i}$.

This framework has been applied to various data sets corresponding to both synthetic and real interaction data sets [109,110] but has yet to be used to study the FcγR-IgG system. The main interest of this framework is its ability to identify the number of pseudo-reactions required to properly explain the sensorgrams by rapidly computing the RCD. Care should be taken when interpreting the RCD, as nodes having a high contribution to the sensorgrams do not necessarily correspond to glycoforms present in high abundance in the injected sample. A somewhat similar approach based on the adsorption energy distribution (AED) has been suggested for the study of SPR sensorgrams by Sandblad et al. [111], but this framework focuses solely on the equilibrium values of the sensorgrams and the equilibrium constant $K_{D,i}=k_{d,i}/k_{a,i}$ of the N pseudo-reactions. This limits its applicability, as reaching equilibrium is not always practically possible.

#### 4.2.4. Conformational Change Model

Another deviation from the ideal 1:1 behaviour consists in an analyte-ligand complex changing conformation after its formation. The complex goes from a first state AL to a more stable state $AL^{*}$. Such a system can be described by two pseudo-reactions in series, each with its own association and dissociation kinetics [112]. It is still unconfirmed whether the application of such a framework to the FcγR-IgG system is appropriate. Hayes et al. [11] found that for FcγRIIIa_V158_ as a ligand, injecting rituximab at a saturating concentration for different durations leads to different dissociation kinetics. For a longer association phase, dissociation was slower. According to them, two reasons could explain this observation. First, a more stable population of rituximab (possibly a different glycovariant) might accumulate on the sensor surface at a higher proportion during the course of the association phase. This comes down to a multiple analyte system, as previously explained. Second, the analyte-ligand complex could undergo a conformational change. As time goes on in the association phase, the proportion of the more stable state $AL^{*}$ would increase, leading to a slower dissociation thereafter. This phenomenon was not observed by Hayes et al. [11] with FcγRIIIa_F158_. Several reports mention the possibility of a conformational change following binding between the Fc region and various Fcγ receptors [15,40,108] but remain inconclusive.

#### 4.2.5. Model Discrimination

As the IgG-FcγR systems are not fully understood, it is not obvious which complex model should be used to describe the sensorgrams. Tiwari et al. [113] suggested a way to discriminate between three complex SPR models: a conformational change model, a heterogeneous ligand model (with two ligand populations), and a bivalent ligand model (one ligand molecule can bind two analyte molecules). These complex SPR models all have two states (two exponential components during both the association and the dissociation phases). As such, the analytical solutions to the ODE systems describing these models are of the same shape and are distinct functions of the kinetic parameters included in each model. Guidelines for the discrimination of these models can be deduced from the analytical solution of each of the models [113]. The authors tested their discrimination method on simulated data. The same group also studied the impact of bivalent analyte binding (one analyte molecule can bind two ligand molecules simultaneously) [114]. The bivalent ligand and bivalent analyte models correspond to 2:1 or 1:2 stoichiometries, respectively. It seems unlikely that these binding mechanisms would apply to the FcγR-IgG systems based on our knowledge of these molecular interactions. Nevertheless, the work of this group offers an interesting mathematical framework for model discrimination, rather than one based on a fundamental understanding of the interaction.

#### 4.2.6. Sensorgram Similarity Score

Karlsson et al. [115] suggested a similarity score to evaluate the resemblance between two SPR sensorgrams. The goal was not to identify kinetic parameters but rather to compare the trajectories of two sensorgrams. This method is particularly interesting for the study of the IgG-FcγR interactions, as they lead to complex sensorgrams, for which modelling is still debated. If two different samples lead to similar SPR signals, one might conclude that both samples are of similar quality (or display the same glycosylation pattern in the case of IgGs or FcγRs). One could compare SPR sensorgrams corresponding to IgG samples from different batches to investigate process repeatability. Moreover, this method could be used to compare a novel biosimilar IgG to its approved reference based on their respective SPR sensorgrams [115].

The method requires duplicates of a reference sensorgram. A mean and a standard deviation are calculated for each time step. A window corresponding to the region mean±A∗st.dev. with A a user-defined scalar is created. A secondary sensorgram that resembles the reference sensorgram should have most of its points within this window. A similarity score can be calculated [115]:

(10) $${\%}_{similarity}={\%}_{points\;in\;window}+{\%}_{points\;out\;of\;window}*\frac{SS_{distance\;window-mean}}{SS_{distance\;points-mean}}$$

where $SS_{distance\;window-mean}$ and $SS_{distance\;points-mean}$ are the sum of squares of the distances between the window borders and the calculated mean and between the points of the secondary sensorgram and the calculated mean.

## 5. Conclusions

The study of the IgG-FcγR binding mechanisms is key to the development of novel therapeutic mAbs with enhanced Fc effector functions. In that endeavour, SPR-based studies have been extensively used to assess the kinetics constants of these interactions. However, the large array of the reported results underlined the complexity of the IgG-FcγR binding. In fact, several studies observed a significant impact of the N-glycosylation of both antibodies and receptors on their interactions. A mix of injected IgG glycoforms in SPR-based experiments would therefore lead to complex kinetics. Over the years, the control and characterization of the glycosylation profile of the IgGs have become essential to consider in the design of SPR experiments with binding to FcγRs. With the N-glycosylation recognized as a critical quality attribute, extensive efforts have been made to better control the final glycans in IgGs production lots. The development of novel cell lines with defined N-glycosylation profiles such as the FUT8 knockout cells (that completely suppress the fucosylation of N-glycans) or the development of predictive models to dictate the culture feed composition hold great potential toward desired, homogeneous and reproducible IgG glycoforms production lots [64,102]. Higher homogeneity among the IgG glycoforms used as analytes in SPR-based experiments could lead to simplified kinetics. More recently, the glycosylation of the FcγR family has also been considered in SPR studies in an effort to further simplify the measured interactions [33].

However, it appears that the scattered results reported in the literature could also be caused by the variability of the experimental SPR protocols. Numerous key parameters must be considered with care in the design of an SPR experiment to obtain accurate data and decrease the complexity in the recorded kinetics. Notably, the immobilization strategy can have a great impact upon the measured kinetic and thermodynamic constants and the reproducibility of the assay. For example, comparative studies have linked artifacts caused by capture antibodies as an immobilization method since it potentially interacts with the Fcγ receptors [13,65]. High flow rates and low levels of immobilized ligands can reduce mass transport limitation artifact [61]. These artifacts significantly impact the resulting data, and it is therefore crucial that SPR-based studies explicitly describe every experimental parameter to enable a rigorous comparison from one study to the other and facilitate the improvement of SPR protocols.

The choice of the analysis model must also be examined to be the most adequate for the interaction under study. Several numerical approaches have been developed in order to offer a rigorous analysis of complex kinetic models. These models enhance the efficiency and application of SPR biosensing by reducing barriers to complex biological interactions. However, several models could fit the same set of sensorgram data. One must understand and fit a model that properly describes the biological behaviour of the interaction. All analysis frameworks described in this review consider only one source of complexity (heterogeneity of the analyte, heterogeneity of the ligand, or conformational change) and assume that all other potential complexity is absent. This assumption does not necessarily hold when studying the IgG-FcγR system. A combination of different factors could cause the complex behaviour of this system, meaning some contributions could remain undetected.

Numerical approaches combined with glycoengineering techniques may thus enable an in-depth understanding of the IgG-FcγR binding mechanisms through SPR-based experiments. Further efforts on the development of novel analytical models as well as algorithms for sensorgram comparison will open the door to more industrial applications of SPR biosensing.

## Figures and Tables

**Table 1:** Impact of IgG quality attributes upon binding to FcγRs.

IgG Quality Attributes	FcγRI	FcγRIIa	FcγRIIb	FcγRIIIa	FcγRIIIb
Deamidation	−	−	−	−	−
Oxidation	None	−	None	None	None
Aggregation	++	++	++	++	++
Deglycosylation	−−−	−−−	−−−	−−−	−−−

The signs defined the effect upon binding as follows −, decreased affinity, +, increased affinity. Adapted from Guijen et al. [26].

**Table 2:** Characteristics of Fcγ receptors.

Receptor	FcγRI	FcγRII			FcγRIII	
		FcγRIIa	FcγRIIb	FcγRIIc	FcγRIIIa	FcγRIIIb
**Structure**	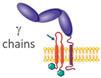	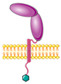	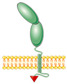	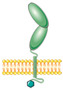	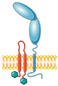	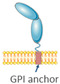
**Signal**	ITAM	ITAM	ITIM	ITAM	ITAM	-
**Polymorphism**	-	R131H	I232T	Q57X	V158F	NA1, NA2, SH
**Affinity for IgG**	High (10^−8^–10^−9^ M)	Low (10^−6^ M)			Low (10^−6^–10^−7^ M)	

## Data Availability

Not applicable.

## Appendix A

**Table A1:** Overview of SPR-based experiments to analyze the affinity between IgG1 and FcγRs.

Receptor	Ligand	Immobilization	*K_D_* (nM)	Analysis Model	References
**FcγRI**	IgG1	Amine	0.12–20.00	1:1 kinetic model	[46,64,116,117]
		Amine	15.40	Steady-state	[10]
		Amine	2.90–41.00	1:1 kinetic model	[35,64]
		Anti-His antibody	0.92–52.00	1:1 kinetic model	[65,118]
	IgG1	Protein A	0.12–0.28	1:1 kinetic model	[119,120]
	IgG1	Goat F(ab′)2 anti-human kappa	0.20–0.23	1:1 kinetic model	[70,121]
		Streptavidin	0.55–3.00	1:1 kinetic model	[14,27]
**FcγRIIa**	IgG1	Amine	690	1:1 kinetic model	[40]
	IgG1	Amine	800–1320	Steady-state	[44,46,116]
		Amine	286	Steady-state	[10]
	IgG1	Protein A	850–972	1:1 kinetic model	[53,119,120]
		Anti-His antibody	4200–6000	Steady-state	[11,118]
	IgG1	Goat F(ab′)2 anti-human kappa	114–203	Steady-state	[70,121]
		Streptavidin	600	Steady-state	[27]
		Streptavidin	480	1:1 kinetic model	[14]
		Kcoil	1542–1916	Steady-state	[13,33]
**FcγRIIb**	IgG1	Amine	3100–3740	Steady-state	[44,68,46,116]
	IgG1	Amine	250–1670	1:1 kinetic model	[40,122]
		Amine	8333	Steady-state	[10]
		Anti-His antibody	6900	Steady-state	[11]
	IgG1	Protein A	1590–2300	1:1 kinetic model	[53,119,121]
	IgG1	Protein A	2100	Steady-state	[123]
	IgG1	Goat F(ab′)2 anti-human kappa	260–1274	Steady-state	[70,121]
		Streptavidin	1660	Steady-state	[27]
		Streptavidin	2700	1:1 kinetic model	[14]
		Kcoil	3949–4670	Steady-state	[13,33]
**FcγRIIIa**	IgG1	Amine	310–850	Steady-state	[12,44,46,71,116]
**V158**		Amine	500	Steady-state	[10]
		Amine	110–244	1:1 kinetic model	[124,125]
		Anti-His antibody	208	1:1 kinetic model	[94]
		Anti-His antibody	89–2166	Steady-state	[76,95,126]
		Anticorps anti-FcγRIIIa	710	1:1 kinetic model	[127]
	IgG1	Protein A	114–280	1:1 kinetic model	[53,119,120,128]
	IgG1	Protein A	136–290	Steady-state	[123,129]
	IgG1	Antibody	7.1–502	Steady-state	[43,74,121]
		Streptavidin	240	1:1 kinetic model	[14]
		Kcoil	402–879	Steady-state	[13,33]
**FcγRIIIa**	IgG1	Amine	5000	Steady-state	[12]
**F158**		Amine	855–1590	Steady-state	[10,65]
		Anti-His antibody	279–1970	Steady-state	[65,95,126]
	IgG1	Protein A	1000–2710	1:1 kinetic model	[53,119]
	IgG1	Goat F(ab′)2 anti-human kappa	13.10–554	Steady-state	[74,121]
		Streptavidin	2290	Steady-state	[27]
		Streptavidin	1300	1:1 kinetic model	[14]
		Kcoil	1912–3852	Steady-state	[13,33]
